# Direct Molding of Luer Connections in PDMS Microfluidic Devices: Comparison with Conventional Interface Methods

**DOI:** 10.3390/mi17091094

**Published:** 2026-09-17

**Authors:** Gina Layedra, Alexander Paolo Vallejo Janeta, Rocio Gimenez, Ramiro Isa Jara, Ana Belén Peñaherrera-Pazmiño, Arianna Mayorga-Ramos, Gustavo Rosero, Maximiliano S. Perez, Betiana Lerner

**Affiliations:** 1 Special Coatings and Nanostructures Engineering (IREN), National Technological University, Buenos Aires 1706, Argentina; gina-layedra@hotmail.com (G.L.); pvallejoj@gmail.com (A.P.V.J.); rocio.gim@gmail.com (R.G.); 2 “Ing. Agr. Alberto Soriano” Graduate School, School of Agronomy, University of Buenos Aires, Buenos Aires C1417DSE, Argentina; 3 Laboratorio de IA “A.M. Turing”, Facultad de Informática y Electrónica, Escuela Politécnica de Chimborazo (ESPOCH), Riobamba EC060155, Ecuador; ramfer.isaj@gmail.com; 4 Centro de Investigación Biomédica (CENBIO), Facultad de Ciencias de La Salud Eugenio Espejo, Universidad UTE, Quito 170527, Ecuador; ana.penaherrera@ute.edu.ec (A.B.P.-P.); arianna.mayorga@ute.edu.ec (A.M.-R.); 5 Mecánica Computacional e Inteligencia Artificial Aplicada (MCIAA), Facultad de Ciencias de La Ingeniería e Industrias, Ingeniería Civil, Universidad UTE, Quito 170527, Ecuador; gustavo.rosero@ute.edu.ec; 6 Department of Electrical and Computer Engineering, Florida International University, Miami, FL 33174, USA

**Keywords:** microfluidic devices, Luer connection, soft lithography, high-pressure flow, leakage resistance, device integration, biofilm, SDG 3

## Abstract

Reliable world-to-chip connections remain a critical challenge in PDMS-based microfluidic devices, particularly under high-pressure flow conditions. In this work, we present a simple, low-cost, and reproducible method to directly mold male Luer cone fittings into PDMS devices during fabrication, without compromising the geometry of microchannels. To evaluate the effectiveness of this approach, we compared the performance of three connection types—PTFE tubing, metal pipes, and molded Luer adaptors—by measuring the infusion pressure at which fluid leakage occurred. Devices with integrated Luer adaptors consistently withstood higher pressures (up to 1555 mbar) with reduced variability across repeated connection cycles, while PTFE and metal connections failed at lower pressures and showed inconsistent leakage behavior. As a biological proof of concept, *Staphylococcus aureus* biofilms were grown in the microfluidic channels under continuous perfusion and subsequently exposed to flow rates of 50, 83.3, and 116.7 µL/min. Image analysis showed an initial biofilm-covered area of 14.22% of the analyzed channel area, while the biofilm areas remaining after exposure to 50, 83.3, and 116.7 µL/min were 1.39, 0.85, and 0.32%, respectively. The final measurement represented a reduction of approximately 97.7% relative to the initial biofilm coverage. The molded Luer connections maintained stable fluid delivery during biofilm cultivation and flow-induced detachment, supporting their use in biological microfluidic applications. Overall, this molding strategy provides a robust and reusable Luer-compatible interface for integrating standardized connections into soft-lithography-based microfluidic platforms.

## 1. Introduction

For more than a decade, microfluidics with lab-on-chip (LOC) devices has become a promising and versatile tool in a wide variety of areas, including cancer research, drug screening, cell culture, and bioremediation, among others. Such devices have been widely accepted and used by the research community in biological and medical fields, mainly due to the advantages of LOCs in terms of miniaturization capabilities, cost-effectiveness, versatility of designs, and promptness in the implementation of improvements [1,2]. In this sense, polydimethylsiloxane (PDMS) has become a cornerstone material in the fabrication of microfluidic devices due to its numerous advantages, including optical transparency, biocompatibility, and ease of fabrication [3,4]. However, one of the persistent challenges in microfluidics is establishing reliable and user-friendly connections between the microscale channels of these devices and the macroscale world of laboratory equipment.

Common methods to connect the microfluidic world to standard fluidic equipment (e.g., syringes and pumps) typically involve the direct insertion and alignment of metal and plastic tubing into the inlet and outlet ports at the external surface of the PDMS layers. When such tubes are directly fitted through the ports of LOCs, there is a high risk of leaking during the experiments, making proper sealing and isolation of the connections essential. The connections are usually sealed and glued to the surface of LOCs using polymer sealants or epoxy glues, though alternatives such as PDMS-molded connectors and reusable plastic connectors have also been considered [5]. However, these traditional approaches pose many constraints to the sustainability of microfluidic devices, including microchannel clogging, increased costs and material consumption, contamination or material incompatibility (especially for biological applications), structural deformation, and fluid leakage [5,6].

In response to these challenges, some authors have proposed the standardization of the connections in “world-to-chip” interfaces [7,8], citing the Luer Lock and Luer Cone adaptors, despite their limited compatibility with most fabrication techniques. According to Temiz et al., the ideal fluidic interconnection should be easy to connect and disconnect, reusable, reliable at high pressures, manufacturable using simple and low-cost techniques, and compatible with commercial tubing and fittings [9]. In this realm, polyvinylsiloxane (PVS), which has been used by dentists as impression material, has been proposed to fabricate microfluidic connectors for PDMS devices for its strong adherence to PDMS and also to avoid thick layers of PDMS in which the tubes or needles can affect sample visualisation within the device. These PVS cylinders are added to a pre-cured PDMS device and ready to use within five minutes, without needing high temperature exposure or the use of additional glues [10]. Moreover, TapeTech solves common setup hurdles by preventing pressure spikes, simplifying priming, and allowing quick installation without disturbing cell culture fluid dynamics. This strategy has been designed for organ-on-chip (OoC) platforms by streamlining how OoCs connect to pumps, and it makes high-throughput testing far more feasible and practical for scaling up complex biological research [11].

To address the resource constraints associated with microfluidic assembly, we propose a simple, highly cost-effective manufacturing methodology that eliminates the need for specialized chemical kits or proprietary bonding reagents, relying solely on polydimethylsiloxane (PDMS). While not natively designed for complex organ-on-a-chip platforms—though adaptable for such advanced architectures in future iterations—this approach provides a low-barrier strategy for low-resource laboratories to reliably interface standard Luer connectors with microfluidic devices. This capability is particularly advantageous for biomedical applications requiring sustained fluid flow, such as in vitro biofilm assays.

Following these design criteria, this study presents a novel, low-cost method for molding male Luer cone fittings directly into PDMS devices without altering the internal microchannel geometry. Its performance was compared with conventional PTFE-tubing and metal pipe interfaces by determining the maximum infusion pressure sustained before visible leakage. As a biological proof of operation, *Staphylococcus aureus* (*S. aureus*) biofilm formation and flow-induced detachment were evaluated in the microfluidic device. Biofilms are surface-associated microbial communities embedded within a self-produced extracellular matrix that promotes persistence through limited antimicrobial penetration, metabolic heterogeneity, slow-growing subpopulations, and reduced immune clearance [12,13]. *S. aureus* is a clinically relevant Gram-positive pathogen frequently associated with persistent biofilm infections on implanted medical devices and tissue surfaces [12,14,15]. Hydrodynamic forces, particularly fluid shear stress, can substantially influence biofilm architecture, stability, and detachment; however, the mechanisms governing flow-induced detachment remain incompletely understood [15,16]. Therefore, this model was selected to determine whether the molded Luer interface could support prolonged biofilm perfusion and subsequent hydrodynamic detachment assays. This biomedical application is aligned with Sustainable Development Goal 3 (SDG 3).

## 2. Materials and Methods

### 2.1. Design and Fabrication of Microfluidic Devices

**Figure 1:**
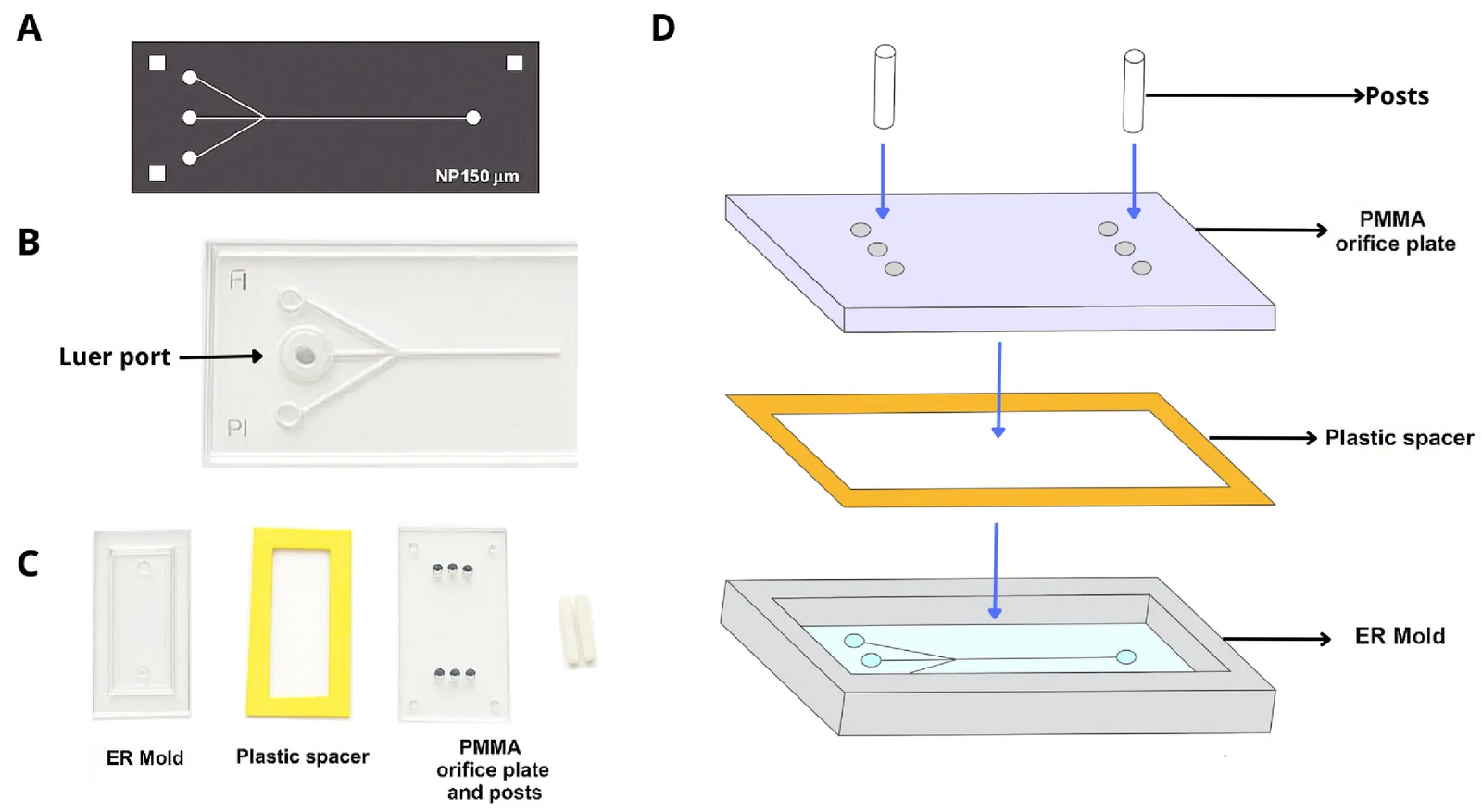
Design and fabrication of microfluidic devices with Luer cone fittings. (**A**) Layout of the microfluidic devices used in this study. (**B**) Image of the PDMS microfluidic device with the Male-Luer cone fittings. (**C**) Molds and custom-made tools used for the fabrication of Luer cone fitting in microfluidic devices. (**D**) Schematic of the assembled system for the male-Luer connection in microfluidic devices.

This study used a microfluidic device comprising a single inlet channel with a channel width of 150 µm which then divides into three outlet channels, using only the one in the middle, while the remaining outlets were sealed (Figure 1A,B). This design was selected to minimize variability introduced by multiple external connections and to ensure that the measured pressure response was dominated by the inlet world-to-chip interface under evaluation. The device layout was designed using LayoutEditor software [17]. A female mold (Fmold) was fabricated using Eastman Kodak’s Flexcel SRH photopolymer and DITR film [18]. The photopolymer sheets measured 1270 × 2062 mm^2^ with a thickness of approximately 1.14 mm. The design transfer process involved exposure to an infrared laser source, solvent washing, and UVA and UVC light exposure, as detailed in our previous publications [19,20].

The Fmold underwent a series of treatments to optimize its surface properties. It was first heated in an oven at 100 °C for 12 h, followed by vacuum treatment at room temperature for 1 h to eliminate residual solvents. Subsequently, the Fmold was cleaned with 70% ethanol solution in an ultrasonic cleaner for 7 min and dried at 40 °C for 10 min. Using the treated Fmold, a male mold (ERmold) was prepared using a commercial epoxy resin and curing agent (Cristal-Tack, Novarchem, Villa Martelli, Argentina) in a 2:1 *w*/*w* ratio. The mixture was sonicated for 14 min to remove air bubbles before being carefully poured over the Fmold and cured at room temperature for 72 h. The ERmold was then carefully separated from the Fmold [21,22].

For the final polydimethylsiloxane (PDMS) microdevice, a Sylgard 184 Silicone Elastomer Kit (Dow Corning, Midland, MI, USA) was utilized. PDMS and curing agent were combined in a 10:1 *w*/*w* ratio following the protocol described by Peñaherrera et al. [2,21,23]. The mixture was degassed under vacuum for 30 min and poured over the ERmold.

After pouring the mixture, a thin plastic spacer was placed at the top of the ERmold to prevent the liquid PDMS from creating a notch when it adheres to the ceiling. A modified PMMA orifice plate into which PMMA posts is then placed, previously molded from syringes due to their conical shape (Figure 1C,D). This additional step in the fabrication of the PDMS replica allowed for the precise molding of a male-Luer cone fitting in the PDMS layer during the curing step. Then, the mixture was cured overnight at 40 °C.

Once curing was complete, the PDMS replica with the integrated male-Luer cone fitting (Figure 1B) was demolded and irreversibly bonded to a glass slide using air plasma treatment. To enhance the glass–PDMS bond strength, the assembled microfluidic device was heated at 40 °C for 1 h, after which it was ready for use.

### 2.2. Comparison of Connection Methods Through Infusion Trials

**Figure 2:**
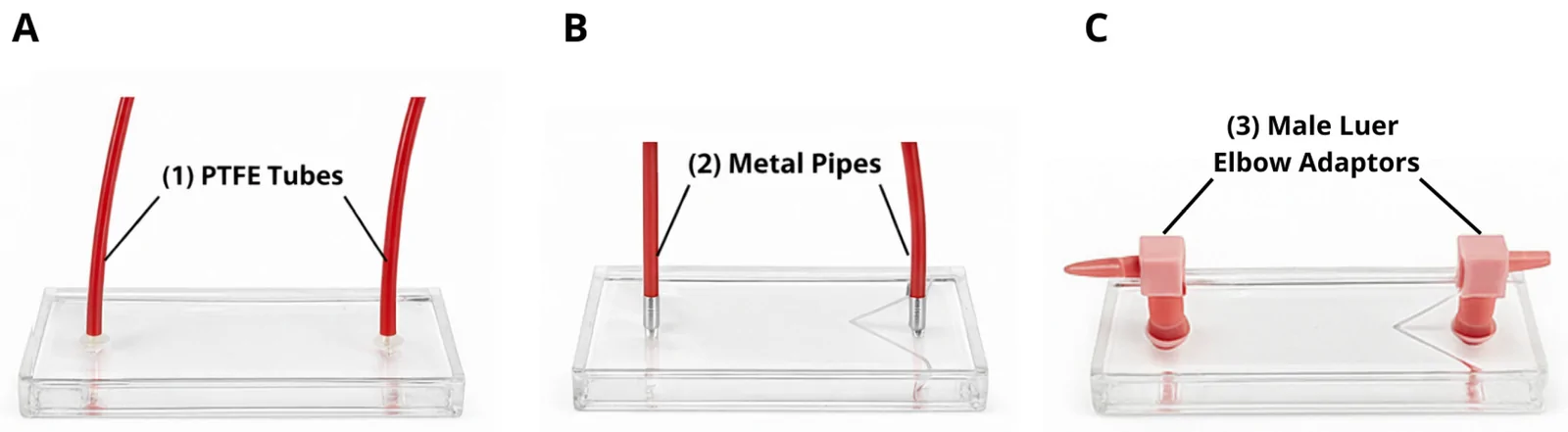
Connection methods tested. (**A**) Direct PTFE tubing connection. (**B**) Metal pipe connection. (**C**) Molded male-Luer elbow adaptors.

Three connection methods for the “world-to-chip” interface were compared using the same lab-on-a-chip (LOC) design, evaluating the infusion pressure sustained until the first leak was observed. The tested connection types included: (1) a direct PTFE tubing connection (inner diameter [ID] 0.8 mm; outer diameter [OD] 1.4 mm) (Diba Omnifit^®^ Tubing, Cole-Parmer, Vernon Hills, IL, USA), in which PTFE tubing was inserted directly into the inlet and outlet ports; (2) a metal pipe connection (ID 0.8 mm; OD 1.0 mm) (Cole-Parmer, IL, USA), inserted between the PTFE tubing and the chip ports; and (3) a molded male Luer elbow adaptors (ID 2.4 mm; OD 4.0 mm) (Ibidi) connecting the tubing to the chip (Figure 2A–C).

For each connection method, three independently fabricated microfluidic devices (Devices A, B, and C) were evaluated as technical replicates. Each device underwent three successive connection–infusion–disconnection cycles (Cycle 1, 2 and 3). For this purpose, all devices were fabricated following the previously described protocol depending on the type of connection. Only the devices incorporating the Luer fitting were prepared using the modified PMMA orifice plate and posts. The Luer interface was not produced by punching a straight inlet, since Luer fittings are based on a conical geometry. Replicating this geometry by molding provides a progressive interference fit, increasing frictional sealing and pressure resistance compared to a cylindrical punched port. The inlet and outlet ports for the direct PTFE tubing and metal pipe connections were created using a 1.2 mm and 1 mm outer diameter biopsy puncher (Integra Miltex^®^ Ted Pella, Inc., Redding, CA, USA), respectively. The punched-port diameter was selected according to the mechanical properties of each inserted component. The rigid metal pipes had an OD of 1.0 mm and were inserted into ports produced using a 1.0 mm biopsy punch. These nominally equal dimensions were selected to avoid the high insertion stresses and potential tearing of the PDMS that could occur if a smaller port were used. In contrast, the 1.4 mm-OD PTFE tubing was inserted into ports produced using a 1.2 mm biopsy punch. The flexibility of the PTFE tubing and the elastic deformation of the PDMS allowed insertion while producing a compressive interference fit around the tubing.

To ensure flow consistency during the infusion, a controlled flow gradient was applied using a syringe infusion pump controlled by in-house open-source software. The syringe pump was programmed to apply flow rates stepwise at 50, 100, 150, 200, 250, and 300 µL/min. Each flow rate was maintained constant for 3 min before the pump setpoint was changed directly to the next value. The complete programmed sequence was maintained until the final setpoint of 300 µL/min, even when the first visible leakage occurred at a lower flow rate, to ensure that the same protocol was applied in all experiments. A dyed 70% (*v*/*v*) ethanol solution was selected as the model test fluid because its relatively low surface tension and favorable wetting behavior facilitate channel filling and the visual detection of liquid penetration through small defects at the connection interface [24,25]. Ethanol solutions are also frequently used for priming and sterilizing PDMS microfluidic devices. It was loaded into a 10 mL syringe during the experiments.

Infusion pressure was monitored using an external sensor coupled to the syringe pump, and pressure values were recorded manually every ten seconds based on the syringe pump display.

### 2.3. Microfluidic Biofilm Formation and Detachment

As a biological proof of concept, *Staphylococcus aureus* biofilms were grown under continuous perfusion and subsequently subjected to a stepwise flow-rate challenge in the same microfluidic device.

First, the *S. aureus* ATCC 25923 from the strain collection (CENBIO, UTE University) was inoculated using the streaking technique on a Luria–Bertani (LB) agar plate. The next day, a single microbial colony was inoculated in rich nutrient Tryptic Soy Broth (TSB) and left overnight at 200 rpm agitation at 37 °C. Cultures were grown for 16–18 h to late-exponential phase and optical density was measured at 600 nm in Cytation 5 multi-mode reader (BioTek, Winooski, VT, USA). Afterwards, the culture was diluted 1:100 into fresh sterile growth medium.

**Figure 3:**
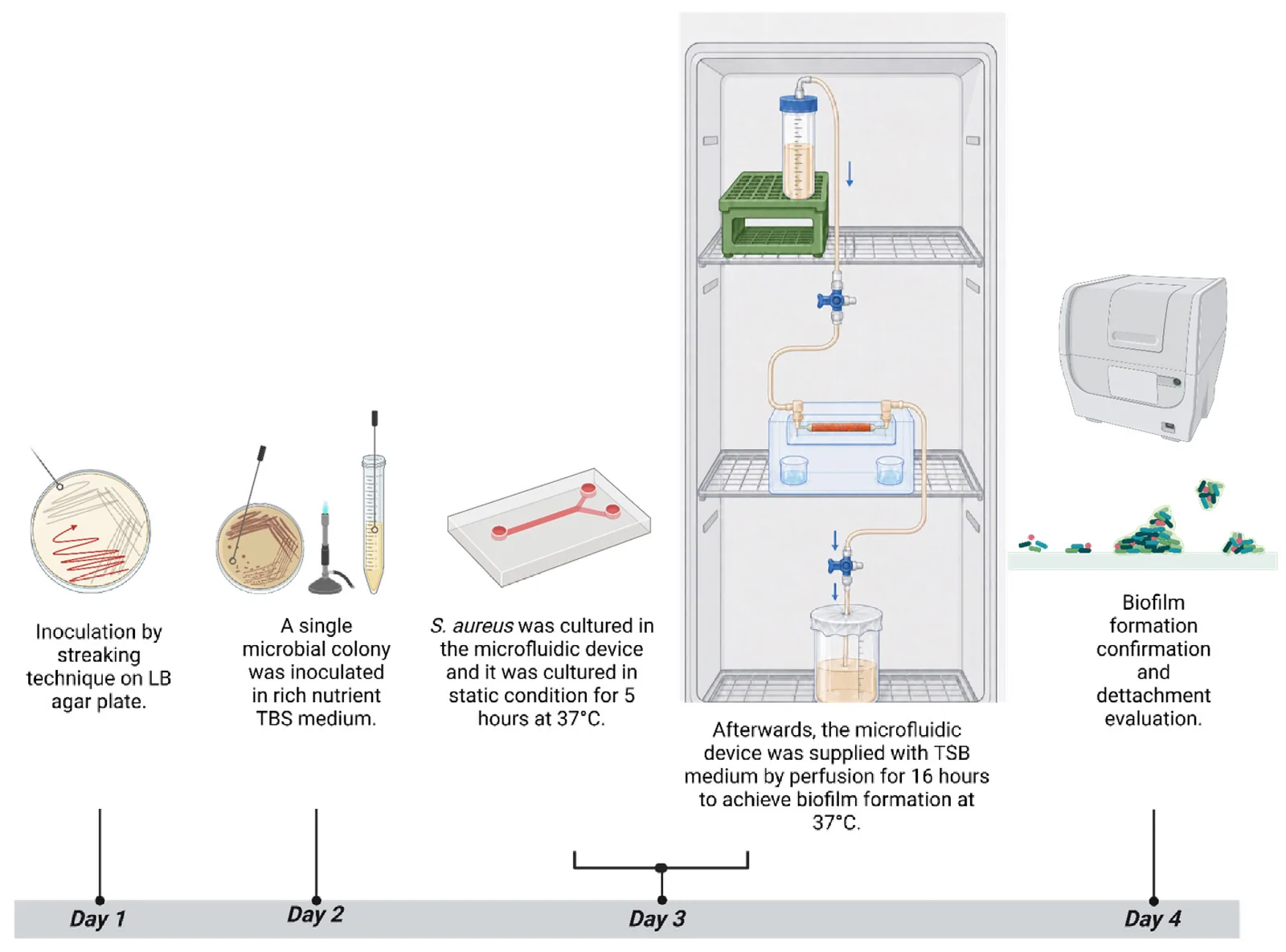
Biofilm assay timeline scheme. Day 1 *S. aureus* inoculation by streaking. Day 2. *S. aureus* inoculation in TSB medium. Day 3. *S. aureus* culturing in the microfluidic device in static conditions inside the incubator for 5 h. Afterwards, TSB medium was supplied through a perfusion system. The experimental setup used for microfluidic biofilm growth under gravity-driven perfusion is depicted. TSB medium flowed from an elevated Falcon tube through the pre-inoculated PDMS device and into a Parafilm-sealed waste beaker. The system was maintained at 37 °C for 16 h, with distilled water reservoirs used to maintain humidity. Day 4. Image acquisition to confirm biofilm growth and detachment assay. Created in Biorender.

This was the initial concentration cultured in the microfluidic device and it was supplied with TSB media by perfusion for 16 h to achieve biofilm formation at 37 °C. The perfusion system utilized to prevent evaporation can be seen in Figure 3. Furthermore, deionized water reservoirs were utilized to provide a humid environment. After 16 h, micrographs of biofilm formation were acquired in Cytation 5 inverted microscope (BioTek, Winooski, VT, USA) with 4× objective to track the complete microchannel and with 20× objective to observe biofilm microstructure.

Once biofilm formation was confirmed, the same biofilm-containing microfluidic device was subjected to a stepwise flow-rate challenge of 50, 83.3, and 116.7 µL/min using the syringe pump SP50 PRO VET (VES, Quito, PI, Ecuador). Each flow rate was maintained for 3 min before progressing to the next step. These flow rates were selected to produce a gradual increase in the hydrodynamic forces acting on the attached biofilm while remaining below the experimentally observed Luer disconnection threshold of approximately 150 µL/min. Accordingly, the selected flow rates represented approximately 33, 56, and 78% of the disconnection threshold. The highest flow rate retained a safety margin of 2 mL/h to reduce the likelihood of connector failure during the biological assay. The 3 min exposure period was selected to provide a standardized acute flow challenge consistent with the step duration used during the mechanical characterization of the connections. Micrographs were acquired before the flow rate challenge and after each 3 min step to quantify changes in the biofilm-covered area.

### 2.4. Image Analysis

The area occupied by the biofilm was measured through image analysis in ImageJ (version 1.54g, National Institutes of Health, Bethesda, MD, USA). Surface coverage was defined as the percentage of the image area occupied by adherent bacterial cells as defined by Shaikh et al. [16]. Images were acquired before and after flow rate exposure for comparison.

To visualize and reconstruct the full geometry of the microfluidic channels across different operating conditions, panoramic images were generated for four distinct channels evaluated at flow rates of 0, 50, 83.33, and 116.67 µL/min. For each condition, 28 individual sequential micrographs spanning from the inlet to the outlet port were acquired and assembled using the open-source software Fiji (ImageJ) equipped with the Stitching plugin. The algorithm was configured to compute the image overlaps using phase correlation with subpixel accuracy enabled to ensure precise spatial alignment between adjacent frames. The final composite images were generated utilizing the linear blending fusion method to smooth intensity transitions across the seams, based on an approximate overlap of 3% between consecutive captures. This approach allowed for a continuous, seamless, and high-resolution comparative documentation of the device performance at each evaluated flow rate [26].

### 2.5. Biofilm Area Percentage Analysis

Panoramic reconstruction of the microfluidic channels, an image analysis procedure was implemented to quantify the biofilm (or bacteria) coverage across the different flow rates. The proportion of the channel area occupied by the bacterial accumulation was determined using Fiji (ImageJ).

Briefly, the composite grayscale images were subjected to background subtraction to correct for uneven illumination, followed by manual or automated thresholding to accurately segment the bacterial regions from the background. Subsequently, the “Analyze Particles” tool was utilized to measure the total surface area covered by the biofilm relative to the total analyzed channel area, allowing the calculation of the percentage occupied area for each evaluated flow rate. Results are summarized in Figure S2.

### 2.6. Statistical Analysis

Values for infusion pressure were presented by device and connection type. Only pressure values where the first leakage occurs were considered for further analysis, then mean leaking pressure values per plug–unplug cycle were calculated and compared among the same connection group and across different connections (Table 1). Significant differences among groups were explored through ANOVA and calculations were made using GraphPad Prism version 10.4.2 (GraphPad Software, Boston, MA, USA).

## 3. Results and Discussion

### 3.1. Infusion Test

Using microfluidic devices with channel widths of 150 µm and a flow rate ramp ranging from 50 µL/min to 300 µL/min (50 µL/min increments) with each step lasting three minutes, three types of connections were tested: PTFE tubing connected directly to the chip, PTFE tubing connected through metal pipes and male Luer elbow adaptors. Although the programmed infusion protocol continued to a final pump setting of 300 μL/min, the maximum operational flow rate reported for each connection corresponds to the highest flow rate sustained before the first visible leakage.

The absolute leakage pressures reported here are specific to 70% ethanol and should not be directly extrapolated to other application-specific fluids. Nevertheless, using the same fluid under identical conditions provides a controlled comparison among the three connection methods.

**Figure 4:**
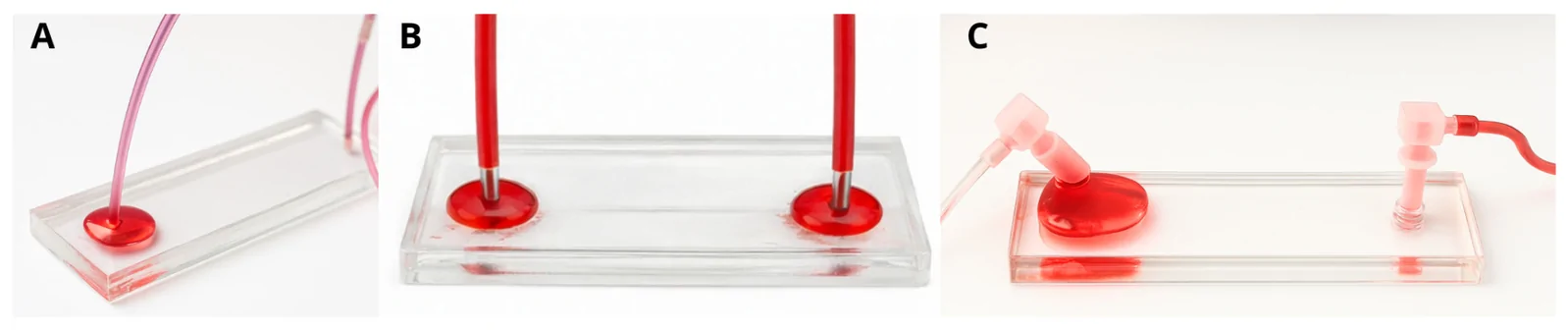
Examples of the leaks observed during the infusion of ethanol 70% in microfluidic devices with different connections. (**A**) Direct PTFE tubing connection. (**B**) Metal pipe connection. (**C**) Molded Luer connection.

Direct PTFE-tubing connections exhibited leakage at 150 μL/min in Device A and during the final seconds of the 50 μL/min step in Devices B and C. Metal pipe connections showed continuous leakage beginning as early as the second minute of the 50 μL/min step. In contrast, molded Luer connections remained leak-free until approximately 150 μL/min, at which point failure occurred abruptly when the interface could no longer withstand the internal pressure (Figure 4). Figure 4C was captured at the onset of this failure and does not represent the normal operating configuration. The programmed sequence was continued after the first visible leakage until either complete disconnection occurred or the maximum programmed setting was reached. Furthermore, due to the tightness of the connections with Luer adaptors, when leaks were not observed, a displacement of the syringe and syringe clamp in the infusion pump was detected, which triggered an alarm that stopped the infusion. This mechanical response was attributed to a higher tightness of the connections leading to stronger retention forces of the Luer fittings, even though the force necessary to unplug this world-to-chip interface was not measured in this work.

**Figure 5:**
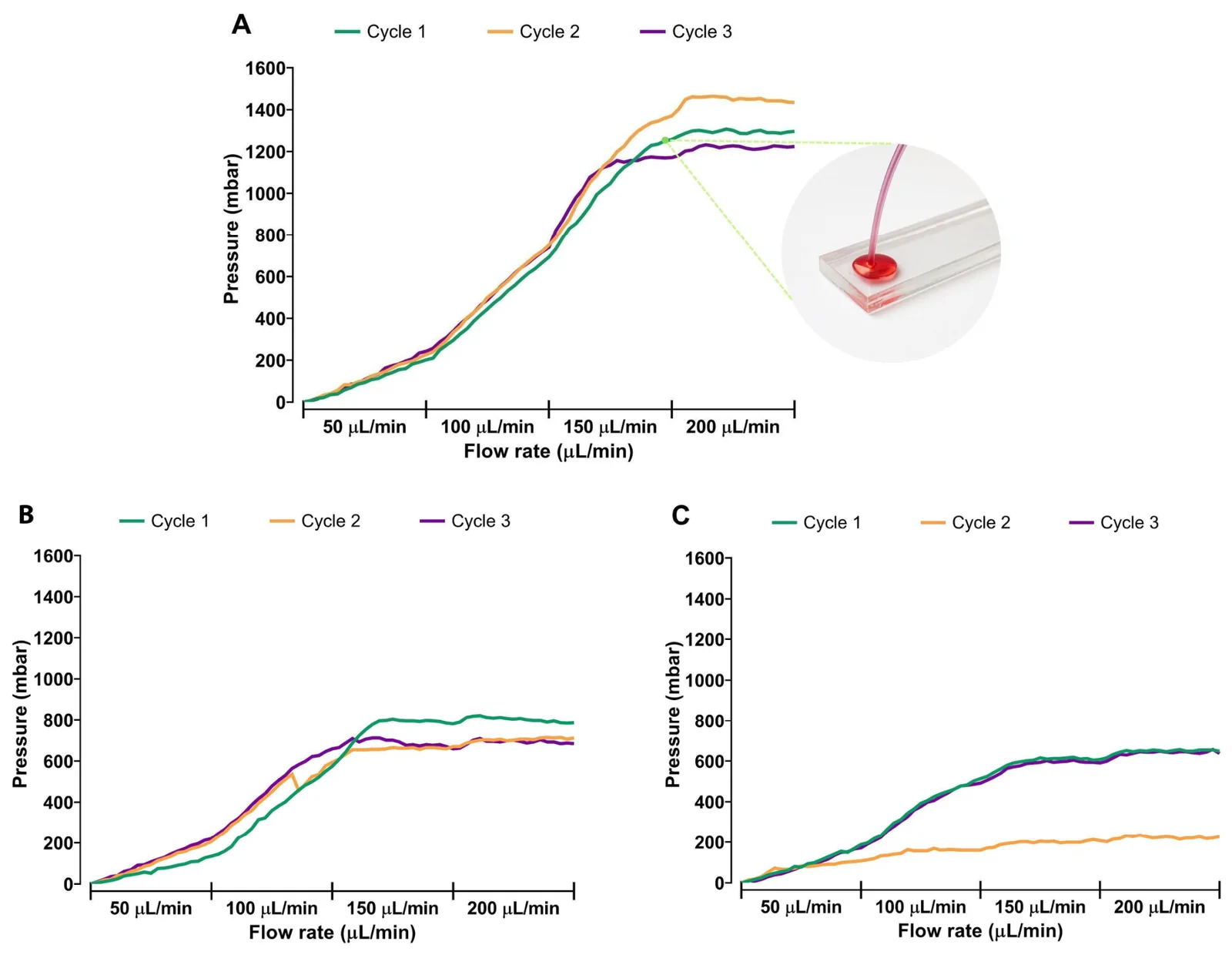
Infusion pressure measurements (in mbar) in microfluidic devices (150 µm channel width) connected to PTFE tubing at inlet and outlet ports. (**A**) Device A with an example of the observed leakage. (**B**) Infusion pressures recorded for device B. (**C**) Infusion pressures recorded for device C. Leakage pressure (LP) is indicated for each device and cycle as the highlighted point in each graph.

**Figure 6:**
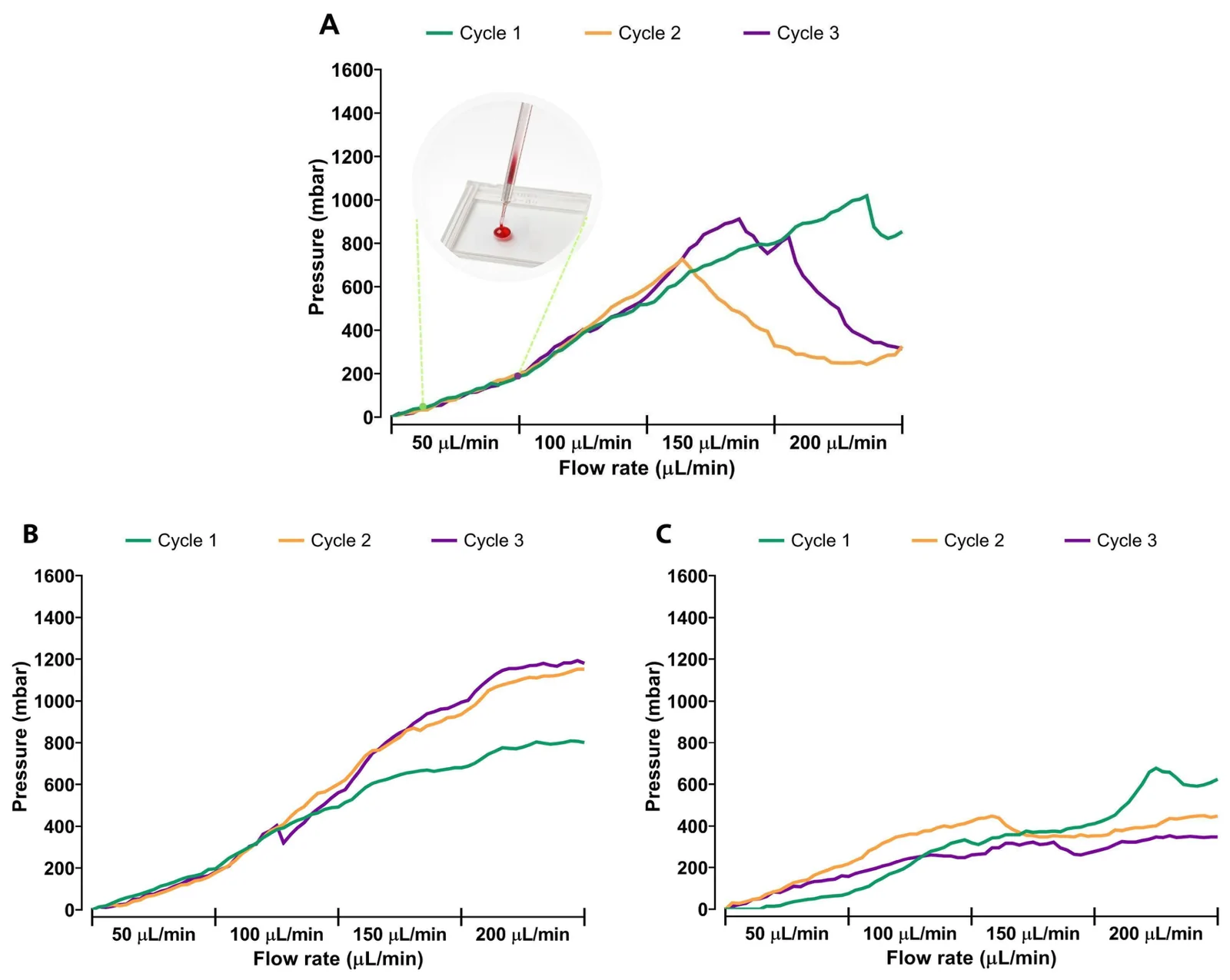
Infusion-pressure profiles (in mbar) obtained for three independently fabricated PDMS microfluidic devices with a 150 µm-wide channel and metal pipe connections at the inlet and outlet. (**A**) Device A, including an example of the observed leakage. (**B**) Device B. (**C**) Device C. Each curve represents one of three successive connection–infusion–disconnection cycles. Differences among panels reflect device-to-device variability.

**Figure 7:**
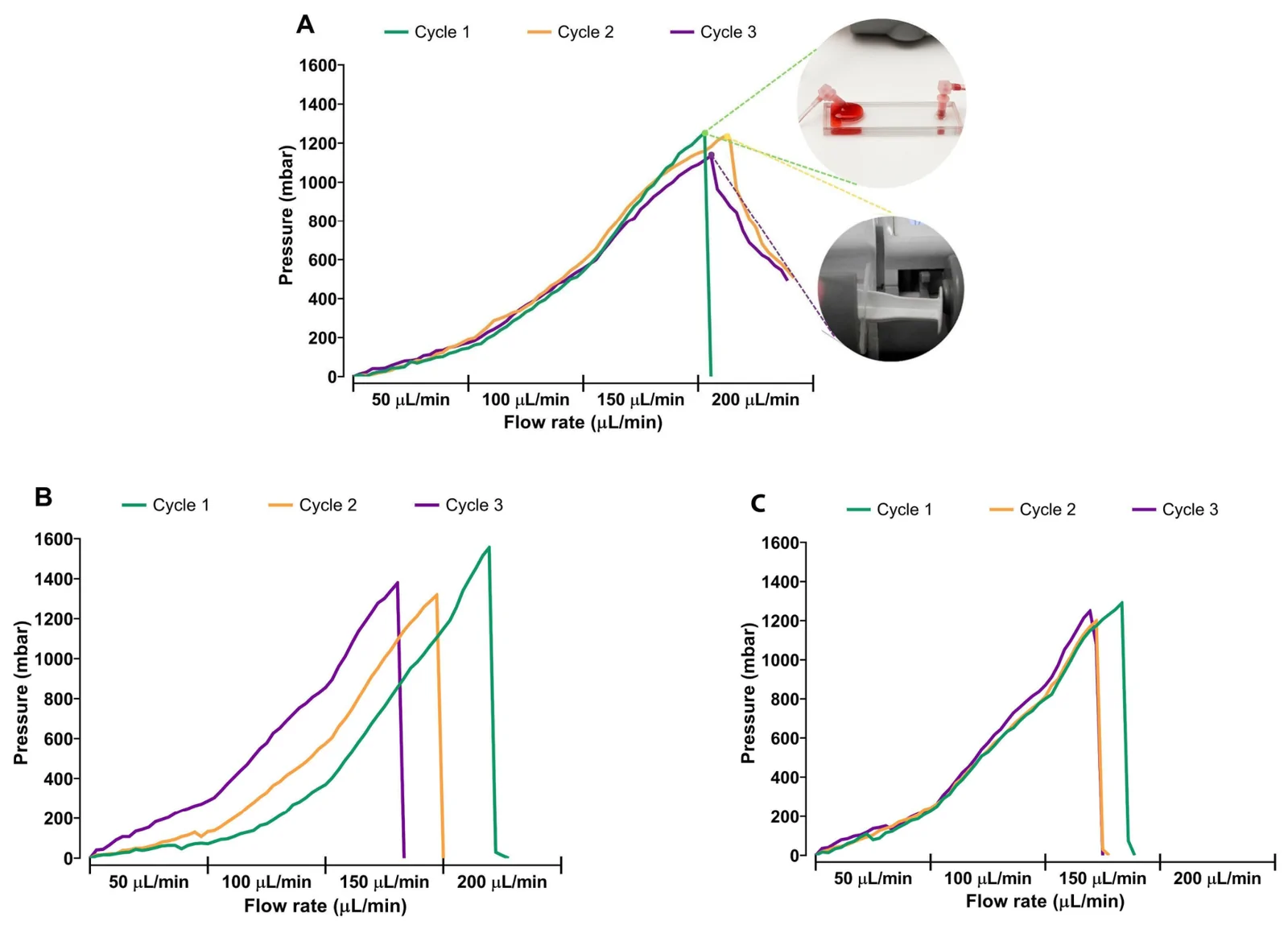
Infusion pressure measurements (in mbar) in microfluidic devices (150 µm channel width) connected to Luer adaptors at inlet and outlet ports. (**A**) Device A with examples of the leakages and flux disruptions observed. When the pressure dropped abruptly and ceased immediately, it was due to the disconnection of the Luer adaptor (e.g., during cycle 1). In contrast, a gradual pressure decrease during the 2nd and 3rd connections was attributed to displacement of the syringe or the infusion pump clamp due to high pressure. (**B**) Infusion pressures recorded for device B. (**C**) Infusion pressures values recorded for device C. Leakage pressure (LP) is indicated for each device and cycle as the highlighted point in each graph.

The pressure flow characteristics of our three distinct “world-to-chip” interfaces were analyzed across flow rates ranging from 50 to 300 µL/min. Infusion pressure values were measured in triplicate across three plug–unplug cycles for each device to confirm our observations regarding the increased strength of the Luer-to-PDMS interface. Indeed, these measurements indicated that non-Luer connections, specifically PTFE tubes (Figure 5) and metal pipes (Figure 6), were unable to bear pressures above 1200 mbar and 200 mbar, respectively. Furthermore, the smooth and consistent pressure curves recorded for devices with Luer adaptors (Figure 7) demonstrated reduced variability and suggests a greater stability of such connections when compared with the devices connected with PTFE tubes and metal pipes.

All three Luer-connected devices exhibit a nonlinear pressure increase with escalating flow rates, characterized by sharp pressure increments at approximately 150–200 µL/min. Among the devices tested, a consistent trend was observed during the first connection cycle, in which the maximal pressure tolerance was the highest. Devices connected by metal pipes exhibited pronounced peak pressures around 150–200 µL/min but exhibited significant variability in maximum pressure values across replicates. In some cases, a significant pressure drop after leakage starts was observed in this type of connection, while in the others pressure remained relatively stable at higher flow rates. Although this connection withstood higher infusion pressures than those connected with metal pipes, leakage was still observed at relatively low flow rates. While pressure profiles were similar across all connection types at lower flow rates, they began to diverge significantly at higher flow rates. An important observation for this last connection is the consistent pressure plateau at approximately 150 μL/min, which suggests critical thresholds in the microfluidic system performance.

To evaluate the stability of molded Luer connectors, we conducted supplementary tests using PDMS microfluidic chips fabricated with increased thickness. These thicker devices were expected to offer greater structural support to the Luer interface. As shown in Supplementary Figure S1, the first connection cycle exhibited the highest-pressure resistance before disconnection occurred. Although a progressive decline in pressure tolerance was observed over the second and third cycles, likely due to mechanical fatigue at the interface, these thicker chips still withstood significantly higher infusion pressures than those observed with PTFE tube or metal pipes connections. This reinforces the advantage of molded Luer adaptors for achieving robust and reusable high-pressure interfaces in PDMS-based microfluidic systems (Figure S1).

**Figure 8:**
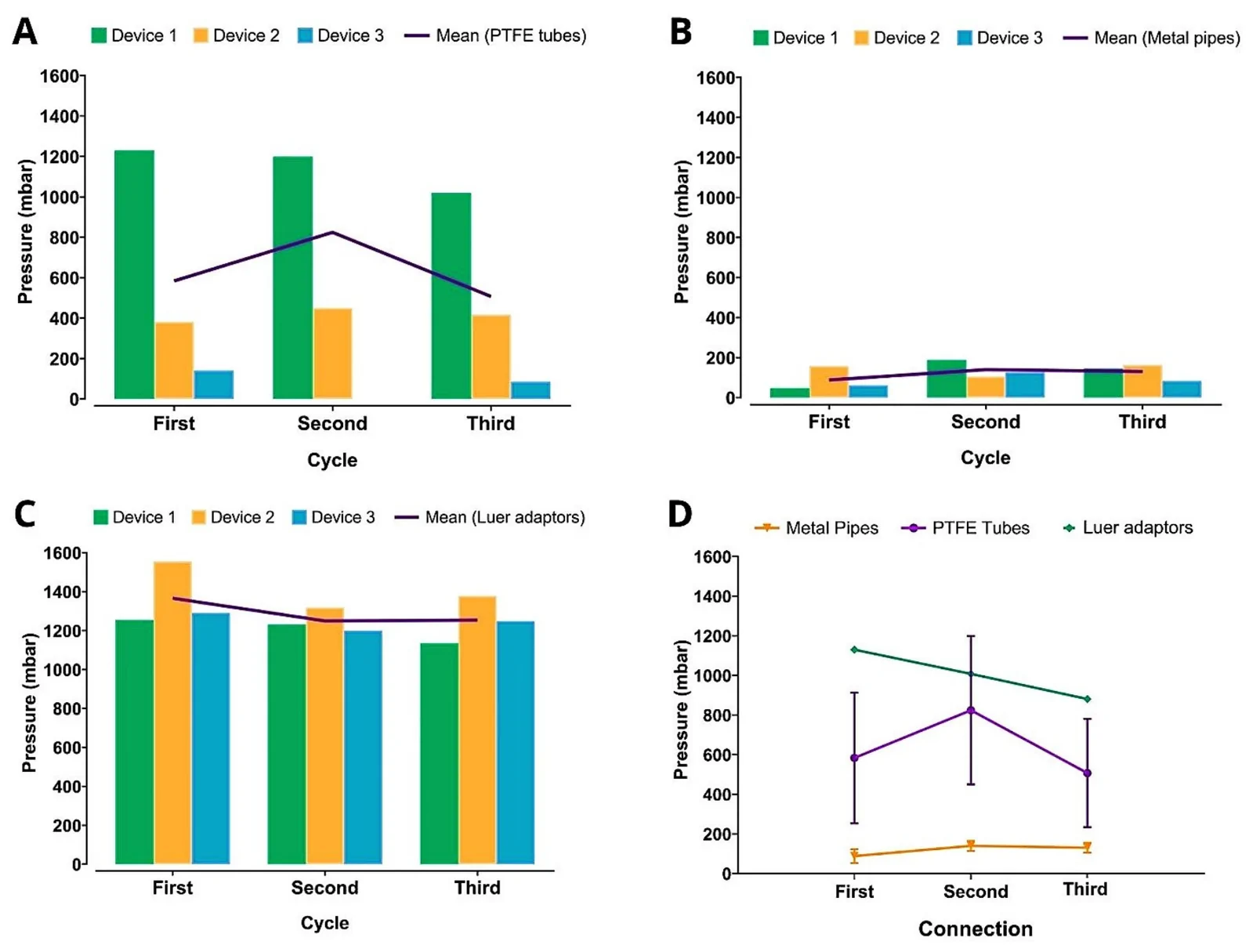
Comparison of leakage pressure values observed at first, second and third cycle with (**A**) PTFE tubes, (**B**) metal pipes, and (**C**) Luer adaptors. Columns represent the pressure value at which first leakage was observed for every device replicate, and lines represent the mean value of the pressure causing the leakage observed during each cycle. (**D**) Mean leakage pressure observed for multiple inputs during the first, second and third cycle. Significant differences (*p*-value < 0.05) were observed when comparing the mean maximum infusion pressures of Luer adaptors vs metal pipes and Luer adaptors vs PTFE tubes during the three cycles. Moreover, significant differences were also present in the overall mean maximum infusion pressures of Luer adaptors vs metal pipes, Luer adaptors vs PTFE tubes, and metal pipes vs PTFE tubes.

Such assumptions are supported by the data in Figure 8 and Table 1, where the maximum pressure values measured for Luer connections are displayed. Maximum pressure values for devices with Luer vary from 1135 mbar (Device A—Cycle 3) to 1555 mbar (Device B—Cycle 1), with the means for each connection ranging from 1366.33 ± 94.90 mbar (Cycle 1) to 1249.67 ± 35.47 mbar (Cycle 2). No significant differences were found among those means, suggesting that no variability is found relative to the maximum pressure that the Luer connections can withstand, which could be translated into a greater stability of connections with our proposed system.

### 3.2. Microfluidic Biofilm Formation and Detachment

The biofilm experiment was included as a proof-of-operation of the complete device rather than as a comparative pressure test. In this assay, the molded Luer interface enabled the long-term perfusion of TSB medium during bacterial cultivation. Biofilm formation was selected as an appropriate biological application because the microchannel design facilitates bacterial attachment, perfusion-based growth, and optical monitoring within the device.

**Figure 9:**
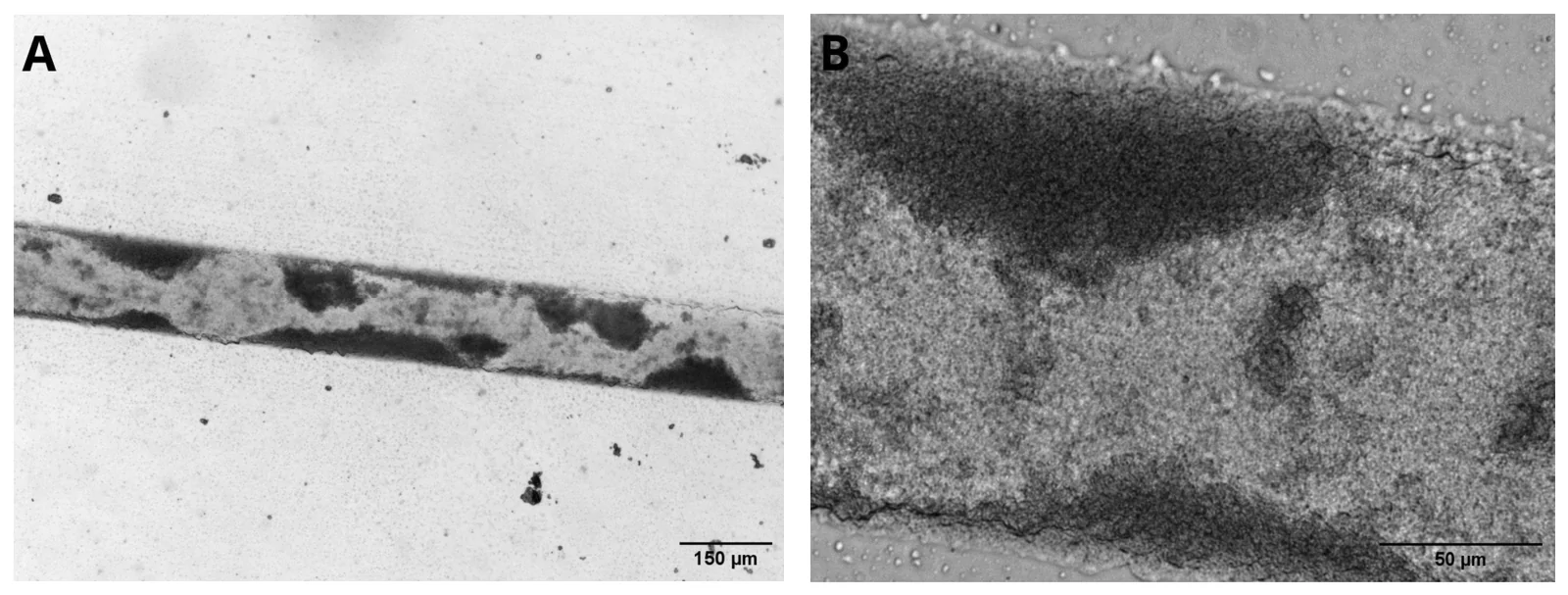
Representative micrographs of *Staphylococcus aureus* ATCC 25923 biofilm formed inside the PDMS microfluidic channel after a 5-h static attachment period followed by 16 h of gravity-driven TSB perfusion at 37 °C. (**A**) Low-magnification view showing biofilm accumulation along the microchannel (4× objective; scale bar: 150 µm). (**B**) Higher-magnification view showing adherent bacterial aggregates and biofilm structure within the channel (20× objective; scale bar: 50 µm).

In the present study, biofilm growth was performed under gravity-driven perfusion, whereas flow rates of 50, 83.33, and 116.67 μL/min were subsequently applied as short-term hydrodynamic challenges to evaluate biofilm retention and detachment. Following the 5-h static attachment period and 16 h of gravity-driven TSB perfusion, *S. aureus* biofilm was observed along the microfluidic channel, with adherent bacterial aggregates and heterogeneous accumulation across the analyzed area (Figure 9).

**Figure 10:**
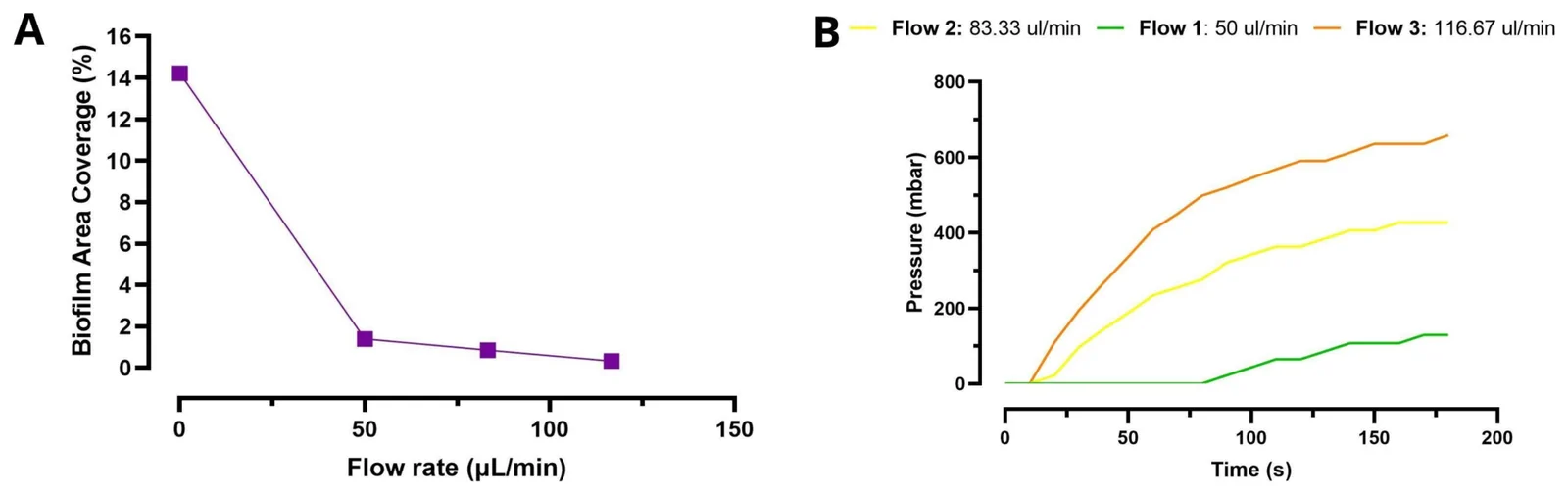
Biofilm-covered area and pressure response during the sequential flow rate challenge. (**A**) Percentage of the analyzed microchannel area covered by *Staphylococcus aureus* biofilm before the challenge (0 µL/min) and after consecutive 3-min exposures to 50, 83.33, and 116.67 µL/min. Biofilm coverage decreased from 14.219% initially to 1.387, 0.852, and 0.322% after the respective flow steps. Because the three flow rates were applied sequentially to the same biofilm in the same microfluidic device, these values represent cumulative flow-induced detachment. (**B**) Pressure time profiles recorded during each 3-min exposure. Increasing the applied flow rate produced progressively higher pressure responses, reaching approximately 130, 420, and 650 mbar at 50, 83.33, and 116.67 µL/min, respectively.

The initial biofilm-covered area was 14.219%. After consecutive 3-min exposures to 50, 83.33, and 116.67 μL/min, the remaining coverage decreased to 1.387, 0.852, and 0.322%, respectively (Figure 10A). Because the three flow rates were applied sequentially to the same biofilm in the same device, these values represent cumulative reductions of approximately 90.2, 94.0, and 97.7% relative to the initial coverage. Concurrently, pressure increased with the programmed flow rate, reaching approximately 130, 420, and 650 mbar, respectively (Figure 10B).

These findings demonstrate that the molded Luer connection supported prolonged gravity-driven perfusion and remained operational throughout the three short-term flow challenges. This operational range was sufficient for the intended biological proof of operation. The operational range of the molded Luer connection was sufficient for the biological proof-of-function evaluated in this study. Previous microfluidic studies have cultivated *S. aureus*-containing biofilms at flow rates of 0.5–1 μL/min and *S. aureus* and *P. aeruginosa* biofilms at 5 μL/min [27,28,29].

## 4. Conclusions

In this study, we evaluated the performance of three commonly used “world-to-chip” connection types (PTFE tubing, metal pipes, and Luer adaptors) when integrated into PDMS microfluidic devices and subjected to increasing infusion pressures. As expected, Luer adaptors demonstrated the highest stability and pressure resistance, withstanding flow rates up to 150 µL/min before leakage occurred. In contrast, metal pipes connections exhibited continuous leakage even at the lowest tested flow rate (50 µL/min), while PTFE tubing connections also leaked at lower pressures than the Luer adaptors. Pressure tolerance measurements further supported these findings, as devices equipped with Luer adaptors reached higher maximum pressure values with lower variability, indicating enhanced connection reliability.

However, the main contribution of this work lies not in confirming the well-known performance of Luer connections, but in presenting a simple, low-cost, and reproducible method to mold male Luer cone fittings directly into PDMS microdevices. This approach does not require external adhesives, machining, or commercial adaptors, and does not interfere with the integrity or geometry of the microchannels. By integrating Luer-compatible interfaces during the molding stage, we significantly improve the durability, reusability, and reliability of microfluidic connections, while maintaining the benefits of soft lithography.

The biological proof-of-concept further demonstrated the operational suitability of the molded Luer interface. *Staphylococcus aureus* biofilms were successfully cultivated under prolonged gravity-driven perfusion, and the same device remained operational during sequential 3-min exposures to 50, 83.33, and 116.67 µL/min. The biofilm-covered area decreased from 14.219% before the flow challenge to 0.322% after the final step, corresponding to a cumulative reduction of approximately 97.7%. Furthermore, this platform permits the assessment of flow rate exposure effect on biofilm detachment. The future perspective would be to use this system to test antibiotic effects on biofilm detachment during perfusion culture. Besides, studies may explore scaling this approach to multi-inlet systems or adapting it for other polymers used in microfluidic device fabrication.

## Figures and Tables

**Table 1:** Leakage pressures (in mbar) measured for the three connection methods. For each connection method, three independently fabricated microfluidic devices (Device A, B and C) were evaluated as technical replicates, and each device underwent three successive connection–infusion–disconnection cycles (Cycles 1, 2 and 3). Values correspond to the pressure at which the first visible leakage was detected. Results are reported individually for each device and as mean ± standard deviation across the three devices for each cycle. No significant differences were observed in mean maximum infusion pressure among the three successive cycles within each interface (*p*-value > 0.05). * Leakage pressure value for device C—second connection with PTFE tubes was excluded due to excessive deviation in comparison with the other trials from the same device.

Connection	Cycles	Device A	Device B	Device C	Mean Leakage Pressure ± SD
PTFE tubes	1	1229	381	141	583.67 ± 571.61
	2	1199	450	*	824.50 ± 529.62
	3	1019	417	86	507.33 ± 473.01
Metal pipes	1	47	157	61	88.33 ± 59.88
	2	188	105	127	140.00 ± 43.00
	3	146	163	83	130.67 ± 42.15
Luer adaptors	1	1254	1555	1290	1366.33 ± 164.38
	2	1232	1318	1199	1249.67 ± 61.44
	3	1135	1378	1249	1254.00 ± 121.58

## Data Availability

The original data presented in the study are openly available in FigShare at DOI: https://doi.org/10.6084/m9.figshare.33755119.

## Supplementary Materials

The following supporting information can be downloaded at: https://www.mdpi.com/article/10.3390/mi17091094/s1, Figure S1: Evaluation of pressure resistance in thicker PDMS chips with integrated molded Luer connectors; Figure S2: Panoramic micrographs of the microfluidic channels showing biofilm accumulation (represented by the dark clusters) under different flow rates from inlet (right) to outlet (left). Panels (a) through (d) correspond to flow rates of 0, 50, 83.33, and 116.67 µL/min, respectively. A clear decrease in biofilm formation and surface coverage is observed as the flow rate increases, demonstrating the detachment and inhibitory effect of higher hydrodynamic shear forces on bacterial accumulation within the channel geometry.
