# Supplementary material for: Direct Molding of Luer Connections in PDMS Microfluidic Devices: Comparison with Conventional Interface Methods
Source: Micromachines (Basel). 2026 Sep 17;17(9):1094. doi: 10.3390/mi17091094 (PMC13609235; doi:10.3390/mi17091094)
Supplement: Supplementary file 1 [file micromachines-17-01094-s001.zip › micromachines-4503880-supplementary.pdf]

Supplementary information.

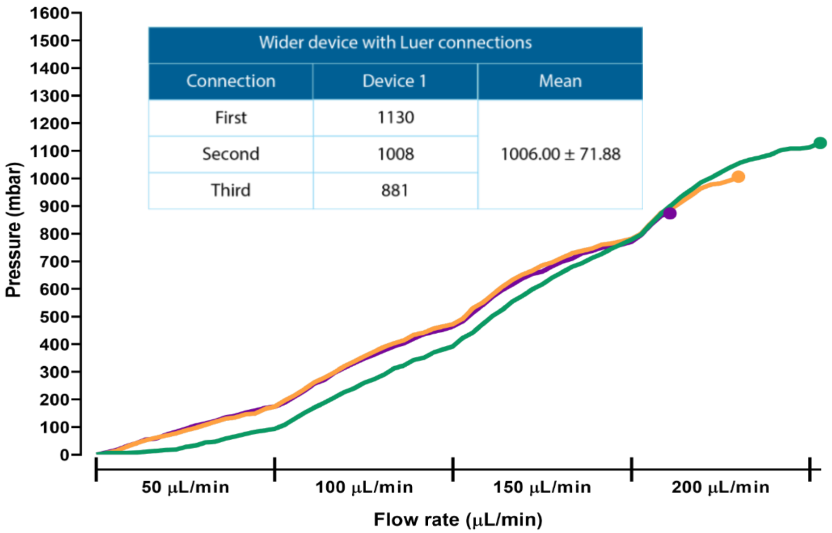

Figure S1. Evaluation of pressure resistance in thicker PDMS chips with integrated molded Luer connectors.

To further explore the mechanical performance of the molded Luer connectors, additional pressure infusion trials were conducted using PDMS microfluidic devices fabricated with increased thickness compared to those described in the main manuscript. The goal was to assess whether enhanced structural support from thicker substrates would improve resistance to disconnection under high-pressure conditions. Each device underwent three successive connection cycles (plug–infuse–unplug), with infusion pressure increased stepwise using the same protocol described in the main study. Ethanol 70% with dye was infused from 50 μL/min to 300 μL/min in 50 μL/min increments, with 3 minutes at each flow rate. As shown in Supplementary Figure S1, the first cycle resulted in the highest maximum pressure before disconnection occurred. A gradual decrease in pressure tolerance was observed over the second and third cycles, likely due to mechanical stress accumulation or deformation at the PDMS–Luer interface. However, despite this decline in performance across repeated cycles, the thicker PDMS chips with molded Luer connectors consistently withstood higher infusion pressures than devices using metal pipes or direct PTFE tubing connections, as reported in the main results. This further highlights the superiority of molded Luer interfaces for achieving stable, reusable, and high-pressure-compatible world-to-chip connections.

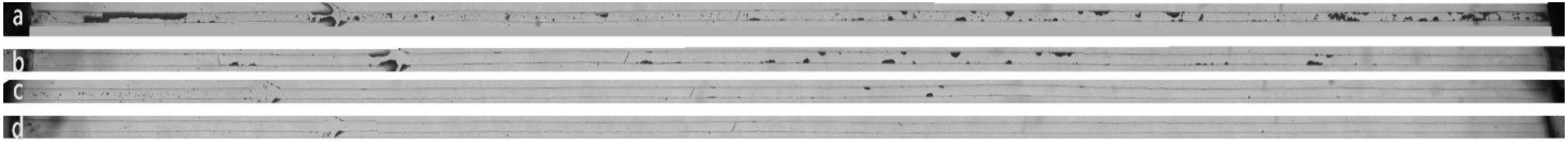

Figure S2. Panoramic micrographs of the microfluidic channels showing biofilm accumulation (represented by the dark clusters) under different flow rates from inlet (right) to outlet (left). Panels (a) through (d) correspond to flow rates of 0, 50, 83.33, and 116.67 μL/min, respectively. A clear decrease in biofilm formation and surface coverage is observed as the flow rate increases, demonstrating the detachment and inhibitory effect of higher hydrodynamic shear forces on bacterial accumulation within the channel geometry.
